# Implementation of the Chief Nursing Officer for England's National Strategy for Nursing Research in Mental Health Service Provider Organisations: A Mixed‐Methods Study

**DOI:** 10.1111/jpm.13169

**Published:** 2025-03-21

**Authors:** Jonny Green, Imogen Byrne, Nicola Clibbens, Carrie‐Ann Black, Geoffrey Dickens

**Affiliations:** ^1^ Mental Health Nurse Research and Education Sussex Partnership NHS Foundation Trust, University of Surrey Guildford UK; ^2^ Cumbria, Northumberland, Tyne & Wear NHS Foundation Trust Newcastle upon Tyne UK; ^3^ Mental Health Nursing Northumbria University, Cumbria, Northumberland, Tyne & Wear NHS Foundation Trust Newcastle upon Tyne UK; ^4^ Nursing for Research and Quality South London and Maudsley NHS Foundation Trust London UK; ^5^ Mental Health Nursing Northumbria University Newcastle upon Tyne UK

**Keywords:** capacity building, leadership, nursing research, psychiatric nursing, research

## Abstract

**Background:**

In 2021, the Chief Nursing Officer (CNO) for England launched the strategic plan for research with the aim of empowering nurses to lead, participate in, and deliver research.

**Aim and Objectives:**

To determine whether mental health service providers have engaged with the CNO's strategy, the extent to which they have developed research leadership roles for nurses, and to identify their related plans.

**Method:**

A convergent mixed‐methods (quantitative plus qualitative) study was conducted. Data were gathered from an online census survey of informants from English NHS mental health service provider organisations, freedom of information requests and web searches. Quantitative data were analysed descriptively, and qualitative data were analysed thematically.

**Results:**

Information was elicited regarding 40/51 relevant provider organisations, *n =* 14 from survey responses and *n* = 26 from Freedom of Information requests. In a subset of provider organisations, dedicated roles for nursing research leadership had been developed, but they lacked consistency in terms of focus, responsibilities and pay grade. We did not identify any specific nursing research strategies; organisation‐wide strategies generally covered all clinical professional groups.

**Discussion:**

The CNOs strategy has had limited influence on providers' research strategy developments. Some provider organisations have actively developed their plans to boost nursing research, but others need to increase their efforts to do likewise or risk missing out on the benefits that accrue to research‐active services, their staff, and service users. We make recommendations for future activity aimed at strengthening nursing research leadership in provider organisations.

**Relevance to Mental Health Nursing:**

Mental health nursing aspires to be an evidence‐based profession, but the contribution made by nurses to the evidence through the conduct and, particularly, the leadership of clinical research has lagged behind that of professional colleagues. Recent attempts to build capacity and capability for research in the mental health nursing workforce have had unclear take‐up and impact. This paper describes how one such initiative, the Chief Nursing Officer for England's strategy for nursing research, has been used in mental health service provider organisations in England.

**Summary**

- What is known on the subject
  ○ Mental health nursing claims to be an evidence‐based profession, but the involvement of this professional group in research, and particularly in research leadership, lags behind that of other professions.
  ○ Active research is commonly associated with indicators of care quality and patient outcomes.
  ○ The Chief Nursing Officer (CNO) for England has published a strategy to facilitate greater nursing research capacity and capability, but its impact has not been evaluated.
- Originality (what the paper adds to existing knowledge)
  ○ This study demonstrates that only a small proportion of mental health Trusts in England have developed infrastructure such as dedicated nursing research leadership posts that will assist in achieving the aims of the CNO's strategy.
  ○ Development of research capacity and capability in mental health nursing has thus far been patchy and lacks consistency across the nation.
- Significance (what are the implications or impacts of the paper)
  ○ The results of the paper demonstrate that mental health provider organisations need to place greater emphasis on delivering the laudable objectives of the CNO's strategy.
  ○ Less research‐active mental health Trusts must develop their own capacity and capability or risk a widening gap between them and more proactive Trusts.

## 1 Background

Worldwide, nurses are the largest graduate professional group working in healthcare organisations (Organisation for Economic Co‐operation and Development 2023), including in UK NHS mental health services where they account for 32% of the workforce (Palmer et al. 2023) and 55% of all registered clinicians (NHS England and NHS Improvement 2022). Since 1941, the most senior advisor to government in England has been the Chief Nursing Officer (CNO), assisted by a number of Deputies, including one with a specific remit for mental health nursing. The CNO is responsible ‘for providing clinical and professional leadership for all nurses, midwives and nursing associates across health and social care in England’ and has objectives which involve recruitment and retention of people with the skills that are required within workplaces that provide opportunity (NHS England n.d.). Those skills and opportunities must reflect the continuing evolution of the nursing role and the enormous influence that research has on nursing practice (Tingen et al. 2009).

There is a growing demand internationally to enable healthcare professionals to support, deliver and lead research across high (Fenlady et al. 2022), middle‐ and low‐income countries (Bilardi et al. 2021). This reflects the positive outcomes achieved in research‐active healthcare organisations more broadly (Boaz et al. 2015), including fulfilment of quality markers (Care Quality Commission 2018), reduced mortality (Ozdemir et al. 2015) and improved recruitment to randomised controlled trials (Borschmann et al. 2014). Additionally, a research‐engaged clinical workforce has the potential to reduce the average 17‐years it takes to translate research findings into clinical practice (Morris et al. 2011).

The frontline role of registered nurses ideally places them in a position to drive forward research that is responsive to challenges in healthcare delivery (Olive et al. 2022); yet, historically, their contribution to research is less than other professions (Henshall et al. 2023). Further, in mental health services, the engagement of all non‐medical professional groups in research activity appears to lag behind their medical professional colleagues (Dickens et al. 2024). In a recent review of UK government‐funded studies, 41% of applicants were medically qualified, 36% were methodologists, while only 8% were nurses or midwives (National Institute for Health and Care Research [NIHR] 2023a). Of concern, nurses were less than half as likely as their medical colleagues to *lead* on research (NIHR 2023a). This deficit appears to be a problem not just in the UK but also internationally (Dickens et al. 2024). Identified barriers to nursing research capacity and capability development include a lack of career pathways, suitable mentors and role models, buy‐in from health services managers (Dorgan 2018) and, crucially, protected time for research activity (Henshall et al. 2023). This issue is not unique to nurses working in mental health; for example, we know that similar problems exist across non‐medical professions in mental health settings too (Dickens et al. 2024). For mental health nurses in particular, however, international variation in the status, role and definition of mental health nursing (Warrender et al. 2023) may contribute to a lack of visibility of mental health nurses within *nursing* research and within *mental health* research more generally (Dickens et al. 2023).

Historic underinvestment in mental health research relative to physical health research (Mental Health Foundation n.d.) has failed to reduce the proportion of the total burden of disease (16%) accounted for by mental ill health (Arias et al. 2022). Relatedly, mental health nurses have historically struggled to develop their collective capacity and capability to contribute to and lead clinically focused research (Butterworth 1991).

Recent investment in England has led to increased funding for mental health research through a £30million sum to ‘rebalance the scale of mental health research’ (NIHR 2021) and £42.7 million through the ‘Mental Health Mission’ (National Institute for Health and Care Research (NIHR) 2023a). Nationally, efforts to grow specific mental health *nursing* research capacity has included publication of a mental health nursing handbook with an emphasis on evidence‐based care (NHS England and NHS Improvement 2022), an improved research career infrastructure for nurses and midwives (NIHR 2022; Menzies et al. 2023) and guaranteed (i.e., funding for nursing and midwifery research) (National Institute for Health and Care Research (NIHR) 2023b). Models supporting research capacity and capability development have identified the need for change and investment at system, organisation and individual levels (Cooke et al. 2018). At an organisational level in healthcare, research capacity and capability development are optimised through professional leadership (Kueny et al. 2015) that supports the development of a research infrastructure including the development of research‐related skills and confidence in the workforce (Gee and Cooke 2018). Achieving an increased volume of nurses engaging with research therefore requires investment in nursing research leadership (Castro‐Sanchez et al. 2020) that is closely aligned to practice (Menzies et al. 2023) and to the clinical‐ and experience‐related outcomes of patients and families (Olive et al. 2022). In response, strategic development in England has involved research capacity building guidance for executive nurses (NHS England 2020), closely followed by improved research career pathways for nurses (NIHR 2023c). In recognition of the significant potential of nurses and midwives to contribute to improving health and wellbeing through research, the Chief Nursing Officer (CNO) for England—in accordance with their role for ensuring continuous nursing role evolution—announced a new strategic plan for nursing research (NHS England and NHS Improvement 2021). This strategic plan sets out a policy framework for enhancing the contribution of nurses to research across the NHS in England across five themes (see Box 1).

BOX 1 **Summary of Chief Nursing Officer for England's strategic plan for research (NHS England and NHS Improvement 2021).**

1. Aligning nurse‐led research with public need—so the portfolios of relevant funders reflect the research priorities of patients, carers, service users, residents, the public and our profession.
2. Releasing nurses' research potential—to create a climate in which nurses are empowered to lead, use, deliver and participate in research as part of their job, and the voice of the profession is valued.
3. Building the best research system—so that England is the best place for nurses to lead, deliver and get involved in cutting‐edge research.
4. Developing future nurse leaders of research—to offer rewarding opportunities and sustainable careers that support growth in the number and diversity of nurse leaders of research.
5. Digitally enabled nurse‐led research—to create a digitally‐enabled practice environment for nursing that supports research and delivers better outcomes for the public.

While, in themes 2 to 5, there is explicit emphasis in the plan on developing nurse leadership in research (NHS England and NHS Improvement 2021), there was, at launch, no available mechanism to benchmark baseline activity or progress on delivery of the CNO strategy. Further, little information exists about awareness of the strategy within mental health provider organisations. In this context, we conducted the current study to clarify these issues.

## 2 Aims and Objectives

The aim of this study was to determine the extent to which recommendations made in the CNO for England's research strategy (NHS England and NHS Improvement 2021) were reflected in the structures, policies and job roles relevant to nurses working in mental health service provider organisations in England. In describing the current landscape, the study aimed to provide a platform for collective learning and development about research‐related leadership roles, their strategic development, and for impact‐evaluation. The specific objectives were to:

- Describe new and existing roles in mental health services that are tasked with leading nursing research for their organisations.
- To understand mental health service perspectives on the current context of building capacity in nursing research, including their awareness of developments such as the CNO's strategy.

## 3 Methods

### 3.1 Study Design

A convergent (QUANT QUAL) mixed‐methods study was conducted. This was a one‐phase design where multiple strategies were utilised to gather and analyse data to answer research objectives. Such an approach is warranted when multiple strategies are required to answer a research problem in a single study (Driessnack et al. 2007). Data collection included (i) a survey questionnaire; (ii) Freedom of Information (FoI) requests which in the UK allow anyone to request to be informed, in writing, as to whether a public authority holds information as specified in a designated request (Freedom of Information Act 2000). This method of data collection is well suited for obtaining information held by public authorities including NHS Trusts (Savage and Hyde 2014); and (iii) a web search for relevant policies and strategies. Data collected was quantitative from closed categorical and multi‐choice items and qualitative from open questions requiring free text responses and from documentary analysis. The purpose of employing mixed methods was to utilise both methodological triangulation (use of more than one method to collect data) and complementarity (gain insight from a greater range of perspectives) (Morgan 2013).

### 3.2 Sample

The sampling unit was mental health service provider organisations in England. Eligibility was determined by the primary function of the organisation being the delivery of mental health services. A census approach was used, providing an opportunity for all relevant organisations to participate. Eligible organisations were identified using the Health Psychology Management Organisation Services A‐Z List of NHS Mental Health Trusts in England (Health Psychology Management Organisation Services 2013) list which was updated and supplemented by the research team to reflect recent changes. In total, 51 mental health provider organisations were identified for inclusion.

### 3.3 Data Collection

First, a survey was conducted among mental health service providers identified in 3.2 above. A link to an online questionnaire, accompanied by a recommendation for completion from the study funder's representative was emailed to the chief nurse. The chief nurse was asked either to forward the survey to the person in their organisation who they considered to be the lead individual for nursing research or, if no such person existed, to complete it themselves. The survey was open for 2 months and reminder emails were sent. Second, Freedom of Information Act (2000) requests were made to organisations who did not respond to the initial survey. Third, a web search for providers' relevant strategies was conducted.

### 3.4 Measures and Materials

Survey. The 37‐item questionnaire was purpose‐designed to address the study objectives and is included as Supporting Information. The tool was not subjected to formal psychometric testing. However, it went through the following developmental process: two of the authors examined the CNO's research strategy and created questionnaire items based on its content; these were supplemented with items based on our study objectives. The draft questionnaire was then circulated among the whole team for comment, including one author who, in her substantive employment in an NHS research position, would have been eligible to complete the study tool, and amended. This process was repeated, and a final draft was sent to a local NHS Trust Director of Research, Innovation and Clinical Effectiveness for comment. Items in the final version covered four domains: (i) the organisation (four items; e.g., services, size and population); (ii) the respondent (five items; e.g., professional background, job title, demographics); (iii) the nursing research leadership role (14 items; e.g., part or whole role; permanent or fixed term role; personal involvement in research activities); and (iv) research strategy in the organisation (14 items; e.g., awareness of CNOs research plan, role of plan in informing research strategy, barriers, facilitators, future plans). Participants were invited to submit copies of relevant job descriptions and strategies with their survey response. Responses were requested in a variety of binary, multiple choice and free text formats.

Freedom of Information requests. Requests were submitted under the FoI Act (2000) to the organisations who did not respond to the survey. Because FoI responses are commonly processed by administrative staff, the requests made through FoI omitted the respondent questions from the survey questionnaire, requesting information from organisational records. Respondents were invited to submit additional material as described in 3.4.1 above.

Web search. A search for providers' relevant strategies using the Google was conducted using ‘Name of Trust’ and ‘research strategy’. If no strategy could be identified on the first page of hits, then ‘nursing strategy’ and ‘strategy’ were also searched. Some organisations provided services other than mental health and therefore the use of ‘mental health’ as a search team was considered too restrictive. Pilot testing of this search strategy on five organisations revealed that relevant content was always on the first page of results and that no more relevant information was located on the subsequent four pages. Relevant strategies were retrieved in full text.

### 3.5 Data Analysis

Except where survey questions were specifically about the individual completing the survey, responses to survey and FoI items were pooled and analysed together because they requested exactly the same information. Descriptive statistical analysis (frequencies and proportions) was carried out on the binary and multiple‐choice elements of survey and FoI data. Data from free text responses to survey and FoI requests were coded independently by two of the team and organised and incorporated into this analysis. Documents (strategies and job descriptions) derived from all three phases of data collection were analysed in a separate exercise. Data was extracted from each strategy as follows: a text search was conducted for the items ‘nurs*’, ‘research’ and ‘clinical academic’. Relevant portions of text were extracted into a Microsoft Word document. These data were treated using the steps outlined by Braun and Clarke's (2006) six‐phase thematic analysis. Coding was conducted by [Author 5] with a sample independently coded by [Author 3]. Coding was inductive and was initially conducted in Microsoft Word. Initial codes were assigned to blocks of text; codes were organised into loose collections of seemingly related themes, and text transferred into tables reflecting initial themes which were agreed by the entire research team. Integration of findings occurred during the drafting and redrafting of the paper by all authors. Two of the authors are highly experienced researchers employed in professorial positions and were involved at all stages of both quantitative and qualitative study design and analysis.

### 3.6 Ethical Considerations

This project constituted service evaluation and did not require ethical approval. However, the project was registered with the host NHS Trust's quality improvement team. A participant information sheet and consent form were included as part of the online survey.

## 4 Results

### 4.1 Quantitative Results

#### 4.1.1 Characteristics of Responding Organisations

Survey responses were received from 14/51 (27.5%) organisations; a further 26 (51.0%) responded to FoI requests (overall response rate 40/51; 78.5%). One response came from a national independent sector healthcare provider and the remainder from NHS Trusts. Responses were made by NHS Trusts in all 15 NIHR (2023c) Clinical Research Network (CRN) Regions (range 1 to 6). NHS organisations served populations of between 155,000 and 4.9 million. Responding organisations provided a combination of mental health (*n =* 39), learning disabilities/neurodevelopmental (*n =* 38), neurology (*n =* 8), offender health (*n =* 29) and substance misuse services (*n =* 19).

#### 4.1.2 Nursing Research Leadership Roles (i) Prevalence and Employment Characteristics

In total, 22 organisations (43%), provided information: of these, *n =* 18 indicated that specific individuals were tasked with such roles while *n* = 4 said there was a team with responsibility. Lead roles were employed on Agenda for Change (AfC) Band 7 up to ‘Very Senior Manager’ including all grade points in between (NHS Employers 2024). One was employed by a higher education institution on an academic pay scale. The four teams that were described as having responsibility comprised multiple roles spanning bands 6‐8b. Of the 18 organisations who reported employing individuals in lead roles for nursing research, 12 said they were permanent appointments; others were fixed term (*n =* 3), secondments (*n =* 2) or honorary (*n =* 1) appointments. Twelve roles involved employees solely funded by the responding organisation while six constituted joint appointments with partner organisations, five with a university partner, and one with a Clinical Research Network and a University. Eighteen different job titles were provided to describe the lead role for nursing research. Eight (44%) included the terms ‘nursing’ and ‘research’, three titles included only ‘nursing’ and five only ‘research’. Two of the nursing titles included ‘professor’.

#### 4.1.3 Nursing Research Leadership Roles (ii) Responsibilities and Reporting

The reporting lines of 22 responding organisations which had an individual or team responsible for leading on nursing research were to: nurse directors (*n =* 11), associate/deputy nurse directors (*n =* 3), heads of department (*n =* 2), management (*n =* 2) or to multiple individuals (*n =* 3) (e.g., Deputy Chief Nursing Officer *and* Director of Research and Development). Organisationally, lead roles sat within NHS research departments (*n =* 12), corporate nursing services (*n =* 4), combined education, research and training departments (*n =* 2) clinical academic teams (*n =* 1), other corporate services (*n =* 1), care group boards (*n =* 1) and a centre of excellence (*n =* 1); *n =* 1 did not respond to this item. Individuals with responsibility for leading on nursing research reported 100% (*n* = 6), 90%–99% (*n* = 2) 50% (*n* = 1), and 40% (*n* = 7) of their role was dedicated to their responsibilities, and *n* = 2 did not respond. Fourteen organisations provided information regarding the job description for the lead role; of these, *n =* 9 reported that it outlined the research‐related responsibilities. Whilst 12 (66%) roles were solely responsible for leading nursing research, others also led on research for other professional groups. One had responsibility for nursing, Allied Health Professionals (AHP) and psychology research. Six lead roles held responsibility for nursing, AHP, psychology and medical/psychiatry research; one of these six roles also had additional responsibility for clinical research practitioners.

Individuals employed in nursing research leadership roles reported responsibility at an organisational level for research training and education (*n =* 12), research planning and policy (*n =* 13), monitoring research quality (*n =* 6) and development of research‐related workforce strategy (*n =* 11), including research capacity development (*n =* 1). Some were responsible for undertaking primary research (*n =* 5) and research supervision (*n =* 8). Additional responsibilities included research delivery roles (e.g., delivery of NIHR portfolio studies and commercial research), data governance, quality control, providing subject expertise, promotion of research within the workforce, and partnership working with external organisations.

#### 4.1.4 Organisational Research Strategy

From all sources, *n =* 47 relevant strategies were identified (*n =* 3 nursing strategies, *n =* 13 research strategies and *n =* 31 organisational strategies). No nursing strategy mentioned research, and no research strategy was specifically a *nursing* research strategy. From the combined survey and FoI responses, *n =* 1 organisation reported having a strategy for *nursing* research, *n =* 1 said that they had one in development, and *n* = 11 did not have one. Seventeen said that nursing research was covered in another strategy, and, of these, *n =* 14 said they planned to evaluate their strategy. Most (*n =* 12; 85.7%) survey responses indicated some familiarity with the content of the Chief Nursing Officer for England's strategic plan: respondents reported that their own strategy had been informed by it to a large extent (*n =* 2), a moderate extent (*n =* 6), a small extent (*n =* 2), or not at all (*n* = 1) while one did not answer. Fourteen (35.0%) respondents to the combined survey and FoI questionnaires reported having a regular organisational meeting where nursing research was discussed. The membership and focus of these meetings were described as ‘nursing only’ (*n =* 4) and ‘multidisciplinary’ (*n =* 10). We also enquired about meeting frequency, chairmanship, terms and conditions and links with national and research bodies, but too few responses were received for analysis.

#### 4.1.5 Job Description Analysis

Nine job descriptions were obtained of persons identified as responsible for nursing research leadership. Job titles and AfC pay bands (NHS Employers 2024) were: Lead Research Nurse/Practitioner (AfC 8a), Research and effectiveness lead (AfC 8a), Head of Nursing and Allied Health Professionals Research (AfC 8b), Managing Partner Research & Development (AfC 8c), Consultant Practitioner, Research & Improvement Clinical Lead (AfC 8c), Head of Nursing Research & Quality (AfC 9c). Two posts did not indicate an AfC pay band: Senior Lead Research Nurse, Clinical Academic Chair in Mental Health Nursing, and one job description did not indicate a job title or AfC pay band. Identified job description elements related to research training provision, research delivery, managing resources/budgets (all *n =* 6), leadership for nurse researchers, clinical leadership, research & development strategy delivery, (all *n =* 5), securing funding, developing research culture (both *n =* 4), advising on research, advocating for research (*n =* 3). The educational levels required were Masters (*n =* 3), and doctoral (*n =* 4). Two did not indicate the required educational level. Four did not stipulate a requirement for published outputs, *n =* 2 stipulated a requirement for published work of sufficient quality for submission to the UK Research Excellence Framework exercise and *n =* 3 suggested that the postholder should publish in peer reviewed journals. One stipulated that the postholder should have experience as a ‘chief, principal, or co‐investigator in research design and conduct’.

### 4.2 Qualitative Results

#### 4.2.1 Survey Respondents View on the CNOs Strategic Plan for Research

Some viewed the strategy as providing a ‘key focus on the importance of research in the nursing profession’, ‘helpful direction’, ‘identified themes’, ‘clear strategy’, an ‘outline for delivery’ and a ‘platform for Trusts to build their own nursing research plans’. In contrast, it was viewed as having been ‘poorly publicised’, ‘lacking in ambition’ and ‘aspirational… but requiring an enormous cultural shift’ for its vision to be achieved.

#### 4.2.2 Views on the Development of Nursing Research in the Organisation

Ten of fourteen survey respondents provided information. Barriers included inadequacies related to protected time for nurses, resources including sufficient staff to facilitate protected time, and monies to support doctoral‐level study and research activity. Nursing research was reported as being viewed as insufficiently important, evidenced in one case by failure to be mentioned in Trust nursing strategy and, in another, by a lack of plans regarding the implementation of what strategy did exist. Other barriers included underdeveloped research‐related knowledge, skills and efficacy within the nursing workforce. Reported facilitators of nursing research included the appointment of an individual/team to lead it. There was acknowledgment that such individuals required adequate mentoring, training and dedicated research time. Further, at an organisational level, developments were needed related to backfill and other methods of resourcing research activity and research‐related education; a need for more meaningful inclusion of research activity in nursing job descriptions, including clearer clinical academic career pathways. In a small number of cases, respondents reported that Trust leadership had committed to developments in these areas.

#### 4.2.3 Nursing Research‐Related Content in Relevant Strategies

Where nursing was mentioned in an organisational research strategy the content was heterogeneous. Some policies recognised a need to provide opportunities to nurses to engage in research activity and/or explicitly stated that there were limited opportunities currently available to some groups to participate in research (e.g., ‘levelling up opportunities for underrepresented groups including nurses’). Some strategies were more specific about the opportunities to be made available including: ‘developing research career pathways for nurses and AHPs’; and an emphasis on early career engagement ‘research experience programme for student nurses.’ The development of research capability through internships, scholarships, training and research skills development were described with some organisations identifying the need for funding provision Collaborative working between health and universities sectors including joint roles and establishment of research units that included mental health nursing were described.

#### 4.2.4 Thematic Analysis of Strategic Documents

This analysis resulted in five themes which appear to capture an interlinked set of processes and aspirational outcomes.

#### 4.2.5 Increasing Opportunity

A mission to increase opportunity was central in many organisational strategy documents. Given the encompassing nature of these strategies, statements were generally inclusive of diverse staff groups but also of patients and the public. The increased opportunities envisaged included increased opportunity for involvement as research participants, for example, in clinical trials, but also in an ever‐increasing range of research management and development activity, including research planning, conduct and implementation.

#### 4.2.6 Workforce

The workforce theme captured the organisational intention to support clinical employees through programmes of research skills improvement and support to undertake academic qualifications. Closely related to this was a commitment in some strategies to research as part of career development and, in turn, this was linked to both staff retention and recruitment. It seemed to be considered that workforce support such as this would be viewed by prospective employees as highly attractive:

‘…the [greater] opportunities for research and development will allow existing employees to improve their skills and further develop their careers right here at [name of Trust], as well as attract high quality clinicians, researchers and other colleagues to join the Trust.’

#### 4.2.7 Culture

In this theme, ‘culture’ extended the focus on individual development to the building of shared values around the desirability of research and related activity per se. Phrases such as ‘embedding research and innovation into practice’ and ‘to be a recognised leader in healthcare research and education by developing a strong research culture across all services’ and a stated commitment to ‘being a learning organisation’ were indicative of organisational commitments. Central to the development of the required research culture was an aspiration to further embed quality improvement activities

‘We will do this by encouraging nursing and AHP led quality improvement programmes, and by championing joint learning across care professions’.

Also key to the development of research culture was the commitment to the develop related infrastructure for example a commitment to ‘renew and further develop the Trust's Clinical Research Facility.’

#### 4.2.8 Partnerships

The theme of partnerships related to a recognition of the need to extend research activities beyond the organisation to achieve research‐related ambitions. For example, the ambition ‘to increase academic partnerships’ aimed ‘to help us provide high quality evidence‐based care through education and research’. For other organisations developing networks across the NHS, wider public services, industry and academia were important to facilitate early evaluation of healthcare innovations. One strategy emphasised partnerships as enabling ‘collaboration and co‐production of research with staff, service users and carers.’

#### 4.2.9 Patient Benefit

Some strategies explicitly acknowledged that the ultimate purpose of their organisational strategy was to enhance patient care and related outcomes: ‘The Trust's research approach for a strong research culture to translate evidence for patient benefit.’ Referencing outcomes specifically: ‘Clinically effective…using NICE guidance, evidence‐based interventions and a range of outcome measures, informed by research.’

## 5 Discussion

This paper describes a mixed methods study about how mental health service provider organisations in England have progressed in the development of roles that are aimed at supporting the development of nursing research and its leadership. The impetus behind the study was to examine progression given the 2021 publication of the CNO for England's nursing research strategy (NHS England and NHS Improvement 2021). Given the relatively short interval since its publication, the aim was not to attribute any developments or otherwise directly *to* the strategy, but to establish a sense of the operational context of nurse research leadership within mental health providers in England.

Survey invitations were sent to the chief nurse in each organisation. Direct responses, despite follow‐up prompts, were made by only a quarter (14/51) of provider organisations. This indicates very limited engagement by nurse leaders in mental health organisations with the nursing research agenda. Further information was generated from FoI requests, analysis of relevant organisational strategies, and job descriptions from roles related to nurse research leadership in mental health services. Most (85.8%) survey responses came from organisations who identified a lead role for nursing research, compared to only 38.4% of the 26 FoI responses, indicating that survey responses represent the progress made by the organisations most engaged with nursing research. One concern arising from the poor response to the survey is that, as new research‐related opportunities arise for nurses as a result of ongoing developments nationally, the most research‐active organisations are better equipped to take advantage of investment and opportunity. Further widening of the gap between research‐intensive organisations and those much earlier in their research capacity and capability development journey could exacerbate inequalities between Trusts with regard to available opportunities for nursing engagement with research and have potential knock‐on effects on clinical outcomes and the recruitment and retention of nursing staff.

Of the responding organisations which employed people in nursing leadership roles, most (12/18; 66%) said roles were permanent and eight (44.4%) said the roles were entirely or almost entirely devoted to nursing research. Six roles represented partnerships with universities and other organisations. Thus, there was evidence that a core of around one third of mental health service provider organisations in England who are sufficiently engaged in research development for nurses have developed roles with a related component; however, only for one in three did that role represent a very significant element of at least one individual's job. While the study may not present an exhaustive picture, the results suggest there is considerable need for improvement. This was also indicated by information about the nature of the extant leadership roles, including a lack of consistency in terms of structure and responsibilities. Among some respondents, clarity was lacking about the leadership responsibilities of the roles. Other variations involved a plethora of job titles, pay grades, whole‐time equivalent status and types of employment contract. It is not currently clear whether such heterogeneity is a benefit or a drawback, but it may by happenstance or design reflect the flexible approach with which the CNOs strategy sets a general course of direction rather than making recommendations or stipulations about how the desired outcomes should be achieved. In addition, where relevant strategies existed, the extent to which they had reportedly been informed by the CNOs strategy varied. This may reflect reports in free text responses that the strategy was sub‐optimally publicised. The job description analysis triangulated survey findings about the diversity/heterogeneity of the lead roles and emphasises the need to consider the benefit of greater standardisation of these roles across organisations.

Specific nursing research strategies were rare, and we were unable to obtain even one example. Organisation‐wide multidisciplinary research strategies were more common, and some made explicit reference to nurses alongside other professional groups as needing special consideration due to their historical lack of opportunity for engagement in clinical research and research leadership. This raises the question as to whether nursing‐specific strategies are warranted. Analysis of the content of organisation‐wide strategies provided richer information about the strategic priorities of organisations in relation to nursing research. The results represent some common themes that should be adopted when addressing nurse research within the organisation, including increasing opportunities for nurses to be involved in research activity, considering research as part of nursing workforce development, addressing the culture to help embed research into practice, utilising partnerships across the wider network outside of NHS organisations, and acknowledging the overarching aim for patient benefit.

Respondents identified barriers to, and facilitators of, the development of nursing research capacity, with issues such as lack of resources and planning being commonly pinpointed in other studies (Dorgan 2018; Henshall et al. 2023). There is a need for resources; however, this should be balanced against the necessity of ensuring that investment delivers in terms of key outputs, which could include grants, papers and clinical outcomes. Further research is required to determine whether wider investment results in improved outcomes, as opposed to outcomes in individual studies.

### 5.1 Implications for Policy and Practice and Future Research

Considering the potential complexity of changes required to meet the CNO strategy objectives, communication systems at a national level to monitor progress and share good practice are needed. In the UK, mental health nurses are well placed to achieve this through three national forums: the national mental health and learning disability nurse director forum (https://mhforum.org.uk/our‐mission), the national mental health nurse consultant forum (Mitchell 2021), and Mental Health Nursing Academics UK (https://mhnauk.org/mhnauk). The CNO should explore the potential for synergy between these forums as an avenue for advancing the CNO's strategy for nursing research.

Individuals holding responsibility for leading nurse research within mental health service provider organisations should be established and linked in with the national lead. Working collectively at a national level could provide a platform for the development of more equal and standardised job roles and responsibilities, and joint work on identifying and delivering research training and priorities. Exercises such as NHS England's own Research demand signalling: mental health nursing can help to guide the formulation of research questions to address known priorities (NIHR 2024).

While the CNO's plan made a reference to ‘gather(ing) evaluative data and assess(ing) progress against expected outcomes’ (NHS England and NHS Improvement 2021, 31) there was no detail as to how this would be achieved. While recent work has seen the development of a tool for use by individual organisations to self‐assess their readiness for supporting research by nurses and allied health care professionals (NHS England 2024a, 2024b) there was also a need to address how progress against the CNO's strategy could be evaluated nationally. The results of the current study go some way to addressing that issue while also highlighting that a number of mental health service provider organisations are likely to be insufficiently engaged to even participate in benchmarking activities.

### 5.2 Study Limitations

This study provides an understanding of the operational context of nursing research leadership and strategy in mental health organisations in England. Limitations include a low response rate to the initial survey; this was partially addressed through FoI requests being sent out to the organisations who did not respond to the survey. Whilst some open response free text was obtained through the survey and by examining strategy documents, the depth and detail of the qualitative analysis were limited. Future research should aim to fill this gap by including qualitative methods to provide richer descriptions of experiences and barriers and facilitators to the implementation of nursing research strategic priorities.

## 6 Conclusion

Recent policy guidance in England provides greater clarity on the research‐related expectations of nurses and allied health professionals from entry level through to consultant level practitioners (National Health Service England 2024a). This has the potential to provide a framework for training and nursing career development pathways that more consistently include research. In turn, this suggests the need for a stronger profile for nursing research within organisational research and nursing strategies. Developments such as the CNOs strategic plan are welcome, but this study suggests that related developments are not distributed evenly across mental health service provider organisations. A greater commitment to develop nursing research careers is still much required.

## Author Contributions

All authors contributed to study conception, design, data collection, data analysis and drafting of the paper. All authors approved the final submitted version of the paper.

## Ethics Statement

The study comprised service evaluation and did not require research ethics approval. However, ethical principles were adhered to throughout.

## Conflicts of Interest

The authors declare no conflicts of interest.

## Data Availability

The data that support the findings of this study are available on request from the corresponding author. The data are not publicly available due to privacy or ethical restrictions.
